# Ultrasound-Assisted Heterogeneous Synthesis of Bio-Based Oligo-Isosorbide Glycidyl Ethers: Towards Greener Epoxy Precursors

**DOI:** 10.3390/molecules24091643

**Published:** 2019-04-26

**Authors:** Corentin Musa, Pierre-Edouard Danjou, Antoine Pauwels, Francine Cazier-Dennin, François Delattre

**Affiliations:** Unité de Chimie Environnementale et Interactions sur le Vivant, Université du Littoral Côte d’Opale, 145 Avenue Maurice Schumann, MREI 1, 59140 Dunkerque, France; corentin.musa@gmail.com (C.M.); danjou@univ-littoral.fr (P.-E.D.); antoine.pauwels@yahoo.fr (A.P.); dennin@univ-littoral.fr (F.C.-D.)

**Keywords:** one-pot synthesis, ultrasound, bio-based, epoxy precursor, isosorbide

## Abstract

The substitution of toxic precursors such as bisphenol A by renewable and safer molecules has become a major challenge. To overcome this challenge, the 12 principles of green chemistry should be taken into account in the development of future sustainable chemicals and processes. In this context, this paper reports the highly efficient synthesis of oligo-isosorbide glycidyl ethers from bio-based starting materials by a rapid one-pot heterogeneous ultrasound-assisted synthesis. It was demonstrated that the use of high-power ultrasound in solvent-free conditions with sodium hydroxide microbeads led for the first time to a fully epoxidated prepolymer with excellent epoxy equivalent weight (EEW). The structure of the epoxy precursor was characterized by FT-IR, NMR spectroscopy and high-resolution mass spectrometry (HRMS). The efficiency of the ultrasound-assisted synthesis was attributed to the physical effects caused by micro-jets on the surface of the solid sodium hydroxide microspheres following the asymmetrical collapse of cavitation bubbles.

## 1. Introduction

Today, the rise in world consumption is leading to an acceleration of the scarcity of resources, and developing alternatives to the finite resources is becoming an important challenge. Thus, the development of the production of renewable or bio-based materials is currently underway in order to substitute materials based on fossil resources [1]. As a part of the development of new eco-friendly materials, bio-based epoxy resins are among the most widely sought-after alternatives [2]. Indeed, due to the versatility of their properties, the epoxy resins are the most popular commercial thermosetting polymers and are found in a large range of applications, such as paints [3], adhesives [4], electronic components [5], and many materials [6]. Unfortunately, bisphenol A (BPA), used as a basic component in the formulation of epoxy resins, is a non-renewable toxic precursor and is being banned [7]. Thus, the major challenge is to substitute BPA for a renewable and less toxic compound with at least equivalent physicochemical properties. In recent years, the quest for new molecular platform molecules for the design of renewable thermosetting materials has attracted a great deal of interest [8], and among these, isosorbide, a sugar-based platform, has emerged as a promising candidate [9,10]. Isosorbide is a bicyclic diol of the isohexide family generated from biomass and qualified by the Food and Drug Administration as “Generally Recognized as Safe” (GRAS) [11]. It provides non-linear optical properties, a thermal stability up to 270 °C, and a high stiffness [12], and it is used as a building block for many bio-based polymers such as polymethacrylate [13], polyesters [14], and non-isocyanate polyurethanes [15,16].

**Scheme 1:**
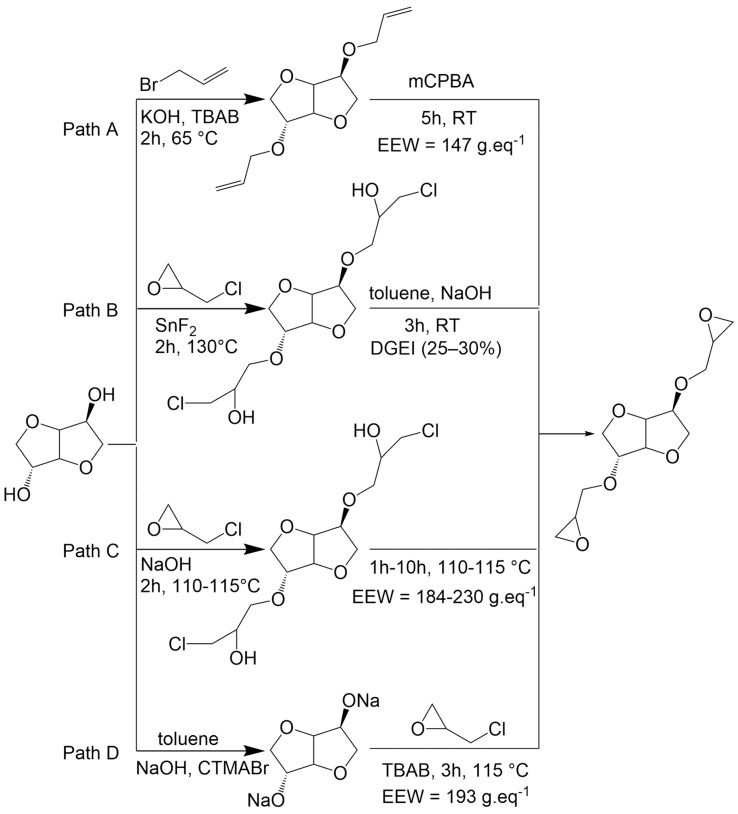
Synthesis of isosorbide glycidyl ethers according to the literature.

Pure diglycidyl ether of isosorbide (DGEI) monomer can be obtained from isosorbide by an etherification reaction with oil-based allyl bromide under phase transfer catalysis conditions followed by oxidation with *m*-chloroperoxybenzoic acid (mCPBA) (Scheme 1, path A) [17]. This route has the disadvantage of generating waste from organic solvents and catalysts, as well as the byproduct m-chlorobenzoic acid which is an antagonist with the atom economy principle. Other routes (path B and D) were developed by employing epichlorohydrin [18,19,20] which can be bio-based from glycerol [21]. However, paths B and D require the use of aromatic solvents (toluene) as well as either metallic (SnF_2_) or organic (CTMABr) catalysts to obtain the desired compound in moderate yields. Greener processes were then developed (path C) using aqueous sodium hydroxide solution. Nevertheless, the use of water in the reaction leads to a partial hydrolysis of epoxy groups, leading to less reactive compounds. Those paths were recently criticized in the literature [20], and it has emerged that there is presently no ideal procedure that allows us to simultaneously obtain excellent yields, a reduced reaction time, pure monomers and green conditions. 

Considering this, and in order to propose a greener route to fully epoxidized oligo-isosorbide glycidyl ethers, this paper reports a new protocol of heterogeneous ultrasound-assisted epoxidation in the presence of atomized sodium hydroxide. This protocol substantially limits the hydrolysis of epoxy rings. In addition, it greatly increases the number of reactive sites. To take into account the principles of green chemistry, the reported synthesis encourages (i) waste prevention, as excess epichlorohydrin can be purified and reused; (ii) atom economy, as the main byproducts are water and sodium chloride; (iii) safer solvents, as no additional solvents were required; (iv) energy efficiency, as the reaction involves the use of an ultrasonic generator for a few minutes compared to the hours of reflux described in the literature; and (v) the use of renewable feedstocks, as isosorbide and epichlorohydrin are bio-based. 

## 2. Results and Discussion

The current route to access oligo-isosorbide glycidyl ether is by reacting isosorbide and epichlorohydrin in the presence of sodium hydroxide at 115 °C, which is the boiling point of epichlorohydrin. In these conditions, although the silent reaction can be done in only three hours, the described optimal synthesis is generally carried out in 12 h at reflux and leads to an incomplete conversion of hydroxyl groups to glycidyl ether accompanied with the partial hydrolysis of the desired epoxy groups. Desired oligo-isosorbide glycidyl ethers were recovered after a filtration step to remove NaCl formed during the reaction followed by a distillation step to remove excess epichlorohydrin and remaining water.

In order to provide more efficient access to oligo-isosorbide glycidyl ethers, it was decided to investigate the impact of ultrasonic activation compared to previously described methods. For this reason, silent and ultrasonic experiments were first performed in homogeneous media using aqueous sodium hydroxide solutions (Table 1, entries 1 and 2). Arbitrarily, the time of reaction in silent mode was reduced from 12 h to 3 h compared to the experiment already described [18,20]. All the results are compared in terms of total masses of oligomers (g), epoxy content (epoxy equivalent weight (EEW); g·eq^−1^) and profiles. Profiles were attributed according to high-resolution mass spectrometry (HRMS) experiments (see below), where Profile 1 presents a high hydrolysis rate of epoxy rings, Profile 2 presents a low rate of epoxy opening with the presence of remaining hydroxyl groups, and Profile 3 corresponds to highly epoxidated compounds.

As shown in Table 1, the conducted syntheses led to a better epoxidation rate for the ultrasonic method with an EEW of 269 g/eq against 284 g/eq for the silent method. In terms of the quantity of product, a better result was obtained by silent synthesis compared to the ultrasonic method (entries 1 and 2). Nevertheless, this latter provided oligo-isosorbide glycidyl ethers in only 15 min, whereas 3 h at reflux were necessary for the silent method. Thus, this first promising experiment revealed that it was possible to effectively use ultrasonic activation for the epoxidation of isosorbide. 

From this first encouraging result, and in order to avoid the hydrolysis of desired epoxy rings as generally reported in the literature [18,19,20], it was decided to substitute the aqueous sodium hydroxide solution with two or four equivalents of solid sodium hydroxide (entries 3 and 5, respectively). Results show in both cases a drastic increase of the mass of products obtained as well as a significantly higher epoxidation rate which is fully consistent with the disappearance of the IR spectra of the hydroxyl stretching vibration band visible between 3470 and 3420 cm^−1^. Thus, for the experiment carried out under ultrasonic irradiation with four equivalents of solid sodium hydroxide, the EEW was found to be 175 g·eq^−1^. The silent reaction was also carried out with two equivalents of solid sodium hydroxide (entry 4) to assess the contribution of ultrasonic activation. In this control experiment, the EEW of the product proves to be relatively close to the one obtained by ultrasonic activation (entry 3) while the recovered amount of oligo-isosorbide glycidyl ethers turned out to be 30% higher by using ultrasound, demonstrating its major contribution to the synthesis.

In order to gain a better insight into the effects of sonication, an experiment was conducted by maintaining the reacting media at 50 °C, as increased temperature has a negative effect on cavitation bubbles and also on sonochemistry. The low mass of the recovered compound suggests that the temperature factor is essential, and the influence of the ultrasound was predominantly of a physical nature [22]. Thus, further experiments were conducted without temperature control with different reaction times. Surprisingly, no time dependence was noticed on the reaction yield from the moment the boiling point of epichlorohydrin was reached.

**Figure 1:**
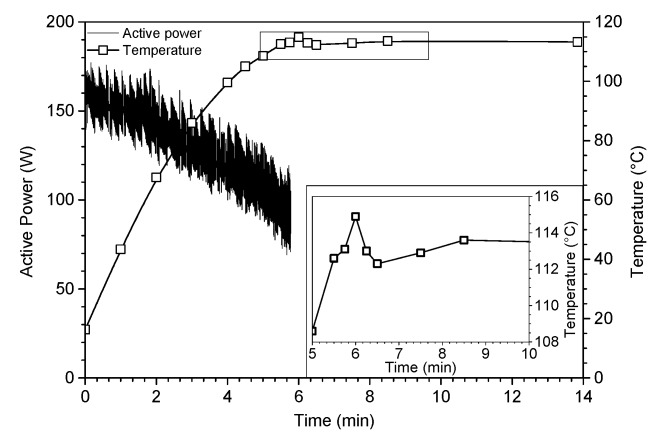
Monitoring conditions (ultrasonics power and temperature) of oligo-isosorbide glycidyl ether ultrasonic synthesis (Table 1, entry 5).

As evidenced in Figure 1, the reflux was reached after only six minutes under ultrasound. From this time, ultrasonic irradiations were stopped and the mixture was allowed to evolve freely with temperature monitoring. Once the ultrasound irradiations stopped, a slight decrease in temperature (~3 °C) was observed before a slight re-increase (~2 °C), which allows us to access a fully epoxidized oligomer of isosorbide. The high decrease of the reaction time is attributed to the physical nature of ultrasound, probably caused on the one hand by the microstreaming in liquid phase and on the other hand by the asymmetric collapse of cavitation bubbles at the microjets in the vicinity of sodium hydroxide microbeads. Indeed, the intensive turbulent motion of the fluid due to high-power ultrasonic waves creates a micrometric velocity gradient acting as a phase transfer agent, inducing a strong molecular stirring at the origin of the acceleration of the reaction rate. In addition, the erosion of sodium hydroxide particles due to the shock wave created upon the implosion of the bubble at the surface of the reactants as well as the sound wave reflection broadcast throughout the medium cause a better diffusion of the sodium hydroxide within the, matrix improving the reactivity. All of these observations highlight the non-purely sonochemical effect, called “false” sonochemistry [23].

**Figure 2:**
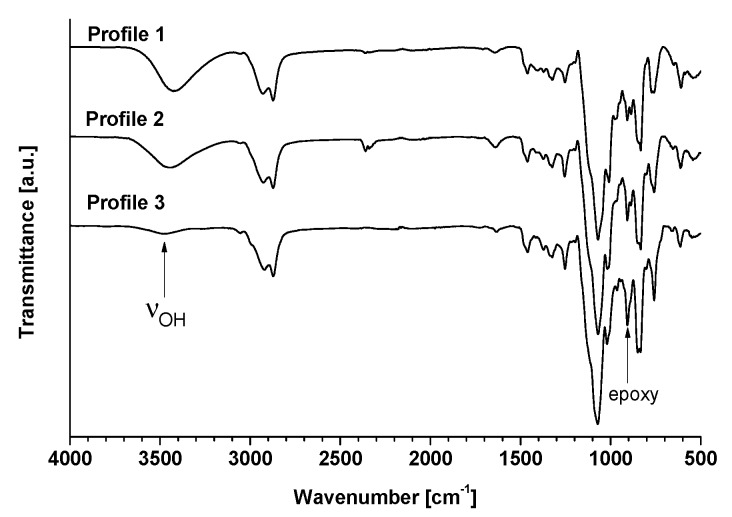
FT-IR spectra of oligo-isosorbide glycidyl ethers.

The efficiency of isosorbide epoxidation was first confirmed by FT-IR experiments (Figure 2). The presence of oxirane rings was principally characterized by the presence of peaks at 1255 cm^−1^ due to the stretching and shrinking of the C-O-C bonds of the epoxy ring and peaks at 950–810 cm^−1^ corresponding to the asymmetrical deformations of the rings. In this zone, two fine and low-intensity peaks are present at 908 and 850 cm^−1^, corresponding to the deformation and stretching of the C-O bonds and to the stretching of the C-O-C bonds, respectively. The broad peak located between 3470 and 3420 cm^−1^ shows the presence of -OH groups. The disappearance of free hydroxyl was confirmed by the drastic decrease of the latter.

**Figure 3:**
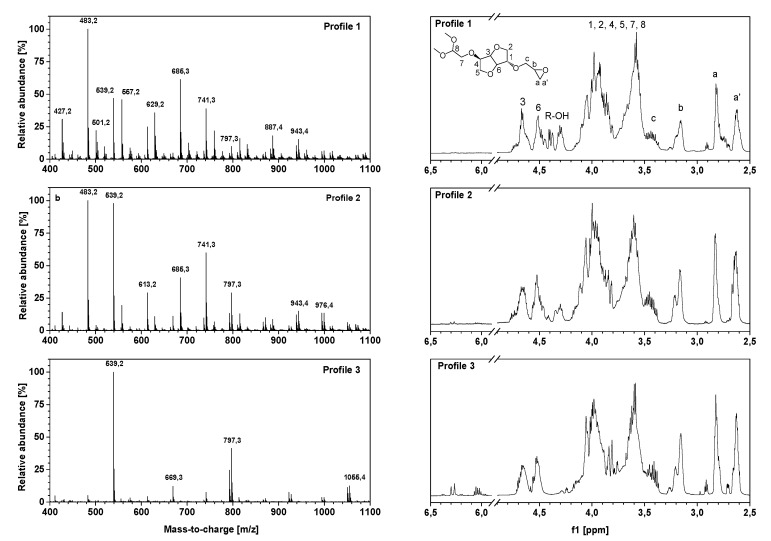
HRMS Q-TOF spectra (left) and ^1^H-NMR (400 MHz, CDCl_3_, 20 °C) spectra (right) of oligo-isosorbide glycidyl ethers for Profiles 1-3 obtained under ultrasonic activation.

The spectroscopic characterization of oligo-isosorbide glycidyl ethers 1–5 by high-resolution mass spectrometry and ^1^H-NMR spectroscopy experiments (Figure 3) allowed us to confirm the efficiency of ultrasonic synthesis and also provided valuable information on the oligomer framework. Three different framework profiles were highlighted depending on the synthesis conditions. Profile 1 corresponds to compounds obtained by using an aqueous solution of sodium hydroxide in silent mode or under ultrasound (Table 1, entries 1 and 2, respectively) while Profiles 2 and 3 match the solvent-free experiments using two and four equivalents of solid sodium hydroxide microbeads (Table 1, entries 3 and 4, respectively). As evidenced by NMR and mass spectrometry, spectra were simplified from Profiles 1 to 3, which is consistent with the yield and EEW previously obtained. 

The presence of glycidyl ether moieties was confirmed by ^1^H-NMR spectra with protons Ha, Ha’, Hb and Hc between 2.5 and 3.5 ppm (Figure 3). Due to the ring conformation and the presence of oligomers, protons H1-H6 of isosorbide were identified as broad signals between 3.5 and 4.2 ppm, as well as by signals between 4.4 and 4.8 ppm that can be assigned to methine protons. Moreover, the peak located at 4.3 ppm confirms the presence of a hydroxyl group on Profile 1 and 2, which is consistent with FT-IR and HRMS. Under ultrasonic activation, the appearance of two peaks at 6.0–6.1 and 6.27 ppm was observed on Profile 3, presumably assigned to a dehydration process leading to the formation of a double bond. 

Otherwise, ^1^H-NMR spectra were used to calculate the ratio R given by the integration of the epoxy ring protons divided by the integration of isosorbide ring protons. This value allowed the evaluation of the degree of substitution of the alcohol functions by epoxy groups; i.e., the higher R was, the more epoxy functions there were per isosorbide cycle. Thus, based on the previously established profiles, the corresponding values were calculated as R_1_= 0.83, R_2_ = 0.98, R_3_ = 1.51 for Profiles 1 to 3, respectively. These increasing values were consistent with the disappearance of the hydroxyl groups in favor of substitution by epoxy functions during syntheses under ultrasound by using sodium hydroxide microbeads.

**Scheme 2:**
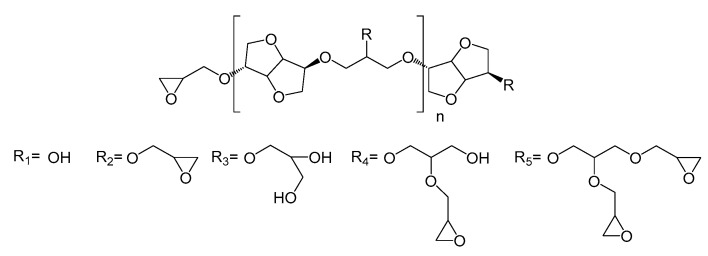
Structures of the oligo-isosorbide glycidyl ethers [R_x_]_n_-R_x_.

The HRMS experiments (Figure 3) show the oligomer distribution for the three profiles of oligo-isosorbide glycidyl ethers depending on the epoxy content. In order to evaluate the synthesis protocols of the oligomers, the different possible structures of isosorbide derivatives were represented in Scheme 2 as well as in Table S1 (see supplementary data). For all spectra, there are no mass peaks before *m*/*z* = 400, indicating that there is no evidence of the presence of a DGEI monomeric structure. 

Owing to HRMS precision, it was possible to finely attribute each major peak as a sodium adduct to structures and to evaluate that most synthesized compounds contain 2 or 3 isosorbides units linked by a 2-hydroxypropyl linker (Table S1, see supplementary data). It is important to notice that no traces of higher oligomers (n > 3) were recorded. An analysis of the Profile 1 mass spectrum allowed us to attribute the following structures (according to Scheme 2) as corresponding mainly to the partial epoxidation of isosorbides units: [R_1_]_1_-R_1_ (*m*/*z* = 427.2), [R_2_]_1_-R_1_ (*m*/*z* = 483.2), [R_2_]_1_-R_2_ (*m*/*z* = 539.2), [R_1_]_1_-[R_2_]_1_-R_1_ (*m*/*z* = 685.3) and [R_2_]_2_-R_1_ (*m*/*z* = 741.3). Interestingly, those peaks had associated peaks at [M + 18] and [M + 2 × 18] corresponding to the hydrolysis of epoxy functions leading to less reactive species. Moreover, in those aqueous conditions, a fully epoxidized compound (dimer) was only represented by the small abundance peak at *m*/*z* = 539.2, while other peaks correspond to product with at least one non-substituted hydroxyl group. Incomplete epoxidation of the isosorbide structure and linker was observed from those results, regardless of the synthesis conditions (i.e. ultrasonic or silent), and this was attributed to the incorporation of water in the reaction media, which led to epoxy group hydrolysis.

To our delight, the substitution of aqueous sodium hydroxide solution by the same molar amount of sodium hydroxide microbeads led to the disappearance of hydrolyzed compound peaks in favor of structures presenting a higher epoxidation rate (Profile 2) compared to Profile 1. Those data agree with IR spectra and allow us to propose the following structures: [R_2_]_1_-R_1_ (*m*/*z* = 483.2), [R_2_]_1_-R_2_ (*m*/*z* = 539.2), [R_2_]_1_-R_4_ (*m*/*z* = 613.2), [R_1_]_1_-[R_2_]_1_-R_1_ (*m*/*z* = 685.3), [R_2_]_2_-R_2_ (*m*/*z* = 741.3) and [R_2_]_2_-R_2_ (*m*/*z* = 797.3). The incomplete conversion of some hydroxyl groups of either isosorbide or 2-hydroxypropyl linked to glycidyl ether was attributed to a lack of sodium hydroxide in the reaction media as epichlorohydrin was added in a large excess. A drastic augmentation of the epoxidation rate of isosorbide oligomers was observed on the mass spectrum by increasing the quantity of solid sodium hydroxide to four equivalents. One can notice that only fully epoxidized isosorbide frameworks were observed with structures corresponding to dimers—i.e., *m*/*z* = 539.2 ([R_2_]_1_-R_2_) and *m*/*z* = 669.3 ([R_2_]_1_-R_5_)—and trimers—i.e., *m*/*z* = 797.3 ([R_2_]_2_-R_2_) and *m*/*z* = 1055.4 ([R_2_]_1_-[R_5_]_1_-R_5_). These results were perfectly consistent with the average of R calculated by NMR experiments, wherein R was estimated at 1.51 for Profile 3, 0.98 for Profile 2 and 0.83 for Profile 1, respectively.

## 3. Materials and Methods 

### 3.1. Chemicals

All chemicals used in this study were used as received without further purification. Isosorbide 98%, sodium hydroxide microbeads and tetraethylammonium bromide 98% were purchased from Acros Organics (Geel, Belgium). Epichlorohydrin ≥ 99% was supplied by Sigma-Aldrich (Saint-Quentin Fallavier, France), glacial acetic acid and perchloric acid 70% were purchased from VWR (Fontenay sous Bois, France). 

### 3.2. Apparatus

Ultrasonic experiments were performed with a sonifier (25 mm diameter horn, 30 kHz, 400 W) from Sinaptec (Lille, France). The sonifier delivered 200 W in continuous mode. The dissipated acoustic power has been evaluated by calorimetric method [24] at 51.5 ± 0.1 W. NMR experiments were recorded on a Bruker Avance III spectrometer at 400 MHz (9.4 T), equipped with a multinuclear z-gradient BBFO probe head. The probe temperature was maintained at 298 K and standard 5 mm NMR tubes were used. The FT-IR spectra (ATR) of precursors were recorded on a PerkinElmer Spectrum BXII spectrometer over the wavenumber range of 500–4000 cm^−1^ with a resolution of 1 cm^−1^. High-resolution mass spectra were recorded on an Agilent 6540 Q-TOF apparatus (ESI+). The EEW values were determined according to ASTM D1652 standard [23] using HClO_4_ in acetic acid as titrant and tetraethylbromide as reagent. Titrations were performed in triplicate. 

### 3.3. Silent Synthesis of Oligo-Isosorbide Glycidyl Ethers 

A series of oligo-isosorbide glycidyl ethers were synthesized according to the previously described etherification method using isosorbide, epichlorohydrin and sodium hydroxide (in aqueous solution or in solid form) solution [18,19]. For this, isosorbide (5 g, 34.2 mmol) and epichlorohydrin (75 mL, 957 mmol) were introduced in a flask fitted with a dean-stark. As referred in Table 1, the synthesis was conducted either by adding the aqueous sodium hydroxide solution (H_2_O/NaOH 50:50 wt.%) drop by drop or by adding the sodium hydroxide microbeads at once. Then, the mixture was stirred under nitrogen at 80 °C for 2 h 30 min and refluxing by up to 115 °C for 30 min. After cooling at room temperature, the mixture was centrifuged in vials at 6000 rpm for 10 min to remove the precipitate. Finally, the water and epichlorohydrin were removed by distillation under reduced pressure. The final product was obtained as a slightly yellow viscous oil.

### 3.4. Ultrasound-Assisted Synthesis of Oligo-Isosorbide Glycidyl Ethers

The ultrasound-assisted synthesis of oligo-isosorbide glycidyl ethers was carried out keeping the same starting materials as in the silent reaction. The ultrasonic probe was placed in the top center of the flask and immersed ~1 cm in the solution. The solution was directly irradiated during ~6 min with an ultrasonic sonifier operating at 200 W in continuous mode. During this time, the temperature of the medium increased to reach the boiling point of epichlorohydrin (115 °C), allowing vaporous cavitations to appear. At this moment, ultrasounds were stopped and the sonifier was removed. At the end of the cooling, the mixture presented a pale-yellow milky appearance and was centrifuged in vials at 6000 rpm for 10 min. Finally, the water and epichlorohydrin were removed by distillation under reduced pressure. The final product obtained was a slightly orange viscous oil.

### 3.5. Characterization of Oligo-Isosorbide Glycidyl Ethers

The determination of the epoxy content was carried out according to the ASTM D1652-11 standard [23]. The EEW is expressed in grams of products necessary to obtain one equivalent of epoxy ring (g·eq^−1^). This titration is based on the in-situ formation of hydrobromic acid (HBr) by the action of perchloric acid (HClO_4_) on an excess of tetraethylammonium bromide (TEAB). For this, 0.2–0.4 g of sample was taken and dissolved in 10–15 mL of CH_2_Cl_2_, 10 mL of a solution of TEAB (25 %), colored with 6 drops of crystal violet indicator solution (0.1 %) then titrated with a solution of perchloric acid (0.1 M). Each assay was carried out in triplicate. 

## 4. Conclusions

The one-pot heterogeneous synthesis of oligo-isosorbide glycidyl ethers was greatly improved by employing high-power ultrasound. This strategy allows a rapid and eco-efficient access to sustainable new materials presenting a high rate of epoxidation as evidenced by FT-IR, HRMS and ^1^H-NMR. Oligo-isosorbide glycidyl ethers prepolymers were obtained in only 15 min, mainly as oligomers of two to three isosorbide units with an improved yield of 30% compared to the silent reaction. Moreover, the ratio of the epoxy ring on isosorbide moiety was superior to 1.5, confirming a high degree of functionalization, which should ensure a high reactivity for a subsequent curing operation. These promising results have been achieved through the combination of solid sodium hydroxide microbeads with the physical nature of ultrasonic cavitation, leading to only anecdotic residual hydroxyl groups on the isosorbide frameworks. Finally, owing to its simplicity and efficiency, this method represents a new, highly efficient synthetic route for the development of a wide range of epoxy precursors.

## Figures and Tables

**Table 1:** Experimental conditions for the synthesis of oligo-isosorbide glycidyl ethers.

Entry	NaOH	Method	Time (min)	Mass (g)	EEW (g·eq^−1^)	Profile
1 ^a^	Aq/2 eq	silent	180	6.4 ± 0.4	284 ± 13	1
2	Aq/2 eq	US	15	5.2 ± 0.2	269 ± 12	1
3	Sol/2 eq	US	15	9.1 ± 0.4	223 ± 10	2
4	Sol/2 eq	silent	15	7.0 ± 0.5	230 ± 12	2
5	Sol/4 eq	US	15	10.3 ± 0.6	175 ± 4	3

^a^ adapted from reference [18]; Aq: aqueous solution; Sol: solid micropearl sodium hydroxide; US: ultrasound; EEW: epoxy equivalent weight.

## Supplementary Materials

The following are available online, Figure S1: HRMS Q-TOF spectra of oligo-isosorbide glycidyl ethers for the different Profiles 1-3 with attribution according to Table S1. Table S1: Structural attribution to HRMS peaks., Video S1: Heterogeneous ultrasound-assisted epoxidation of isosorbide.
