# Supplementary material for: Ultrasound-Assisted Heterogeneous Synthesis of Bio-Based Oligo-Isosorbide Glycidyl Ethers: Towards Greener Epoxy Precursors
Source: Molecules. 2019 Apr 26;24(9):1643. doi: 10.3390/molecules24091643 (PMC6539049; doi:10.3390/molecules24091643)
Supplement: Supplementary file 1 [file molecules-24-01643-s001.zip › molecules-492736-SI.pdf]

molecules

MDPI

Type of the Paper (Article)

Supplementary data for

Ultrasound-Assisted Heterogeneous Synthesis of Bio-Based Oligo-Isosorbide Glycidyl Ethers: Towards Greener Epoxy Precursors

Corentin Musa, Pierre-Edouard Danjou, Antoine Pauwels, Francine Cazier-Dennin and François Delattre*

Unité de Chimie Environnementale et Interactions sur le Vivant, Université du Littoral Côte d'Opale, 145 Avenue Maurice Schumann, MREI 1, 59140 Dunkerque, France

* Correspondence: delattre@univ-littoral.fr

Received: date; Accepted: date; Published: date

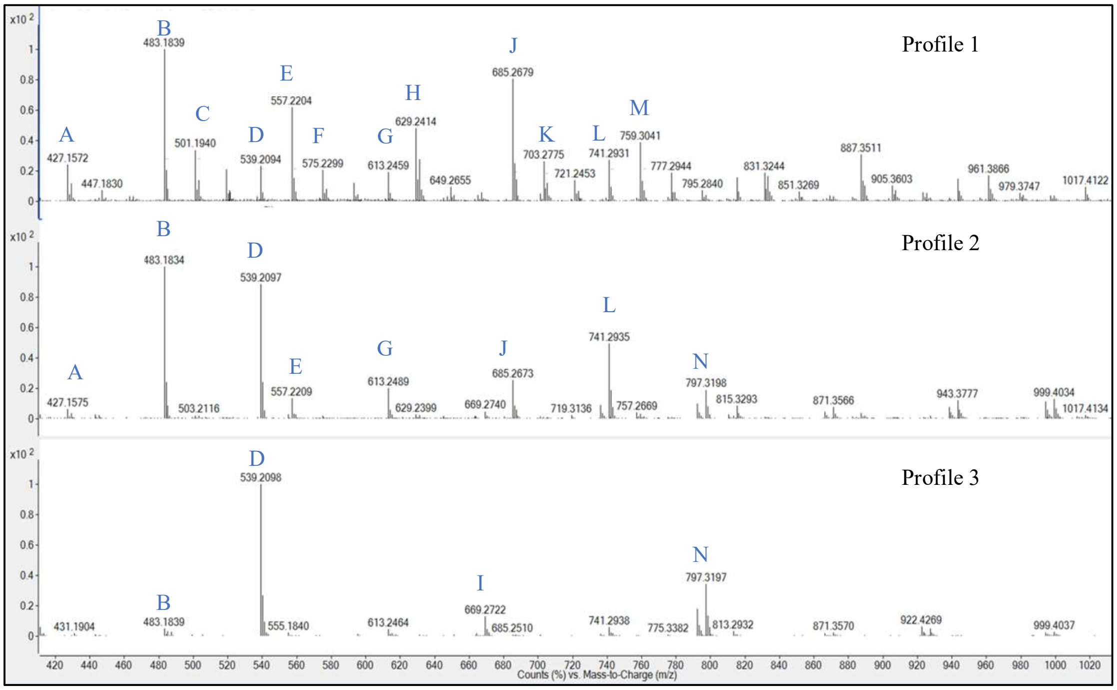

Figure S1. HRMS Q-TOF spectra of oligo-isosorbide glycidyl ethers for different profiles 1-3 with attribution according to Table S1.

Table S1. Structural attribution to HRMS peaks.

Entry	Exact mass calculated for Na$^+$ adduct and mass found	Proposed structures. Red: desired epoxy groups; green: incomplete reaction; blue: epoxy hydrolysis
**A**	Chemical Formula: $C_{18}H_{28}NaO_{10}^+$ Exact Mass Calc.: 427.1575 **Found: 427.1572**	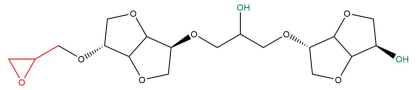
**B**	Chemical Formula: $C_{21}H_{32}NaO_{11}^+$ Exact Mass Calc.: 483.1837 **Found: 483.1839**	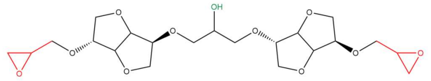
**C**	Chemical Formula: $C_{21}H_{34}NaO_{12}^+$ Exact Mass Calc.: 501.1942 **Found: 501.1940**	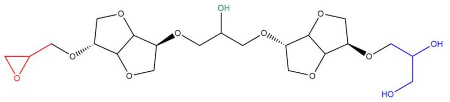
**D**	Chemical Formula: $C_{24}H_{36}NaO_{12}^+$ Exact Mass Calc.: 539.2099 **Found: 539.2094**	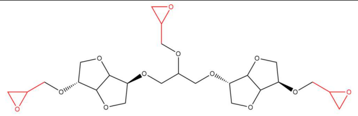
**E**	Chemical Formula: $C_{24}H_{38}NaO_{13}^+$ Exact Mass Calc.: 557.2205 **Found: 557.2204**	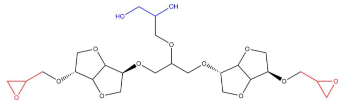
**F**	Chemical Formula: $C_{24}H_{40}NaO_{14}^+$ Exact Mass Calc.: 575.2310 **Found: 575.2299**	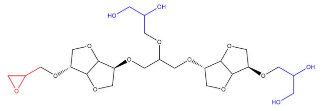
**G**	Chemical Formula: $C_{27}H_{42}NaO_{15}^+$ Exact Mass Calc.: 613.2467 **Found: 613.2459**	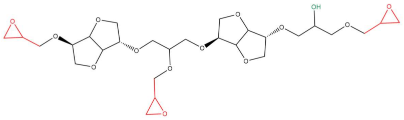
**H**	Chemical Formula: $C_{27}H_{42}NaO_{15}^+$ Exact Mass Calc.: 629.2416 **Found: 629.2414**	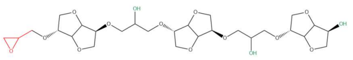 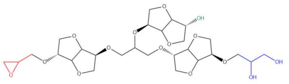
**I**	Chemical Formula: $C_{27}H_{42}NaO_{15}^+$ Exact Mass Calc.: 669.2729 **Found: 669.2722**	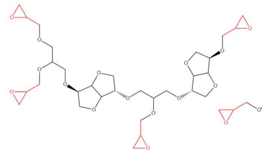 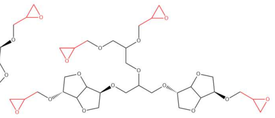
**J**	Chemical Formula: $C_{30}H_{46}NaO_{16}^+$ Exact Mass Calc.: 685.2678 **Found: 685.2679**	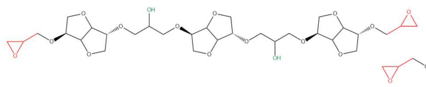 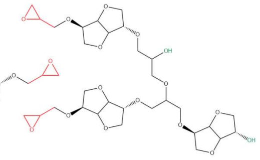
**K**	Chemical Formula: $C_{30}H_{48}NaO_{17}^+$ Exact Mass Calc.: 703.2784 **Found: 703.2775**	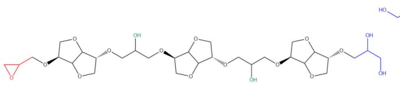 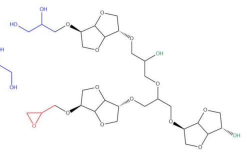

**L**	Chemical Formula: $C_{33}H_{50}NaO_{17}^{+}$<br>Exact Mass Calc.: 741.2940<br>**Found: 741.2931**	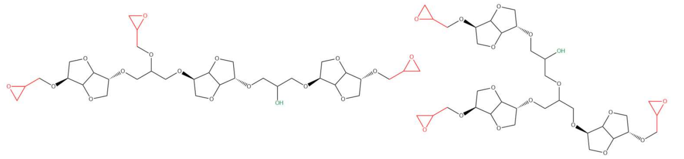
**M**	Chemical Formula: $C_{33}H_{52}NaO_{18}^{+}$<br>Exact Mass Calc.: 759.3046<br>**Found: 759.3041**	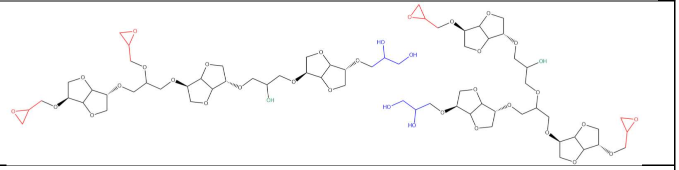
**N**	Chemical Formula: $C_{36}H_{54}NaO_{18}^{+}$<br>Exact Mass Calc.: 797.3202<br>**Found: 797.3197**	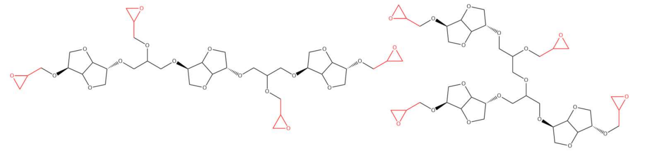
